# Supplementary material for: Genetically stable CRISPR-based kill switches for engineered microbes
Source: Nat Commun. 2022 Feb 3;13:672. doi: 10.1038/s41467-022-28163-5 (PMC8813983; doi:10.1038/s41467-022-28163-5)
Supplement: Supplementary file 1 — Supplementary Information [file 41467_2022_28163_MOESM1_ESM.pdf]

Supplemental Information

Genetically stable CRISPR-based kill switches for engineered microbes

Austin G. Rottinghaus[1+], Aura Ferreiro[2,3+], Skye R.S. Fishbein[2,4], Gautam Dantas[2,3,4,5,6*], and Tae Seok Moon[1,6,**]

[1]Department of Energy, Environmental and Chemical Engineering, Washington University in St. Louis, St. Louis, United States
[2]The Edison Family Center for Genome Sciences & Systems Biology, Washington University School of Medicine, St. Louis, MO, United States
[3]Department of Biomedical Engineering, Washington University in St. Louis, St. Louis, MO, United States
[4]Department of Pathology and Immunology, Washington University School of Medicine, St. Louis, MO, United States
[5]Department of Molecular Microbiology, Washington University School of Medicine, St. Louis, MO, United States
[6]Division of Biology and Biomedical Sciences, Washington University in St. Louis, St. Louis, United States

[+] These authors contributed equally to this work.

* Correspondence
Gautam Dantas
Email: dantas@wustl.edu

** Correspondence and Lead Contact
Tae Seok Moon
One Brookings Dr., Box 1180
St. Louis, MO 63130, USA
Tel: +1 (314) 935-5026
Email: tsmoon@wustl.edu

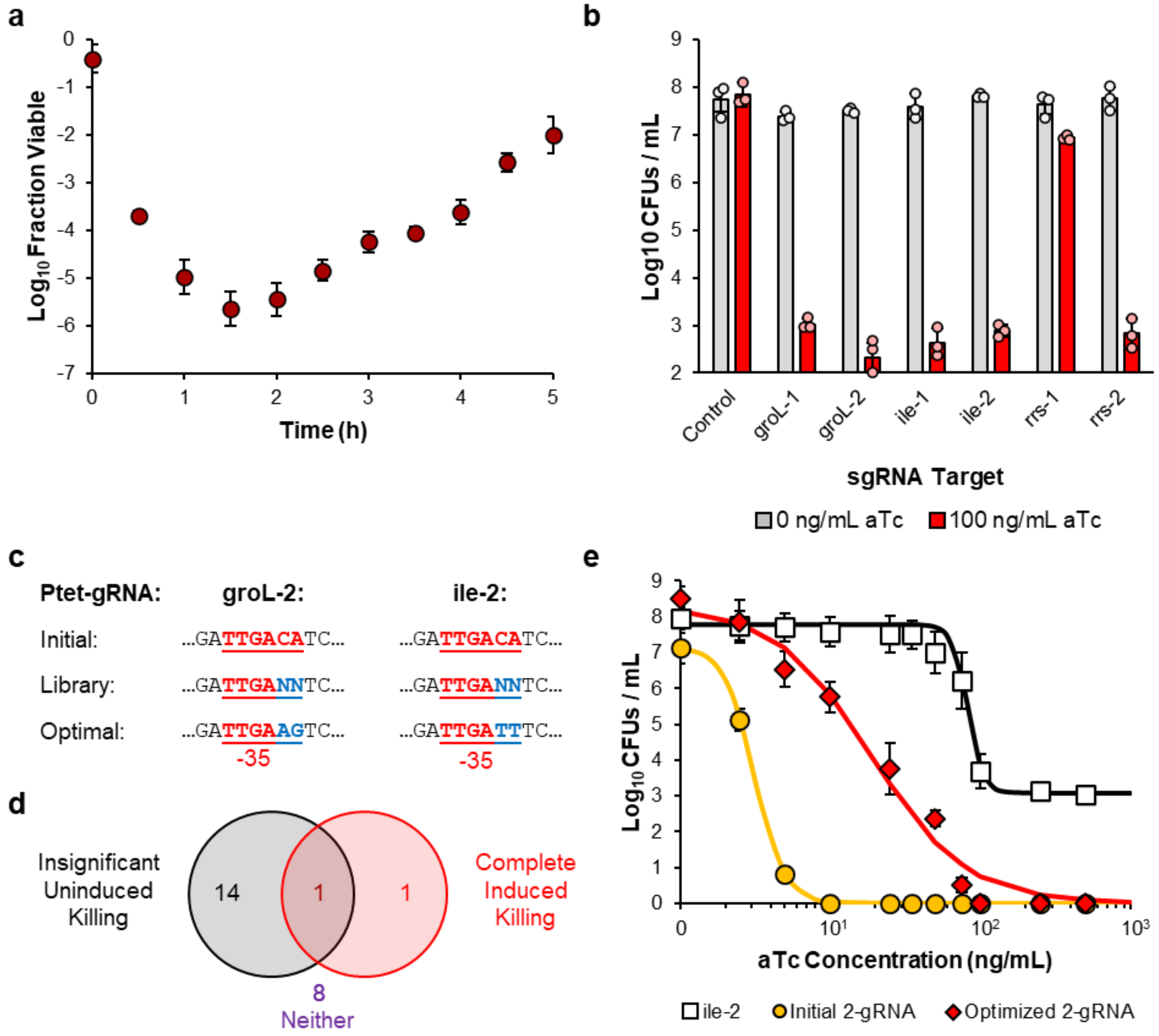

Supplementary Fig. 1 Characterization of single-gRNA and 2-gRNA kill switch strains, related to Fig. 1

(a) Log_{10} fraction viable time course for ile-2 gRNA expression plasmid in wild-type EcN with the Ptet-*cas9* expression plasmid.

(b) Log_{10} CFUs for the no gRNA control and six gRNA expression plasmids in EcN with four genome-integrated P_{tet}-*cas9* expression cassettes.

(c and d) P_{tet} promoter library (c) design and (d) screening results for optimizing the 2-gRNA kill switch plasmid. (d) Number of library variants that demonstrated insignificant killing in response to 0 ng/mL aTc, complete killing in response 500 ng/mL aTc, both, or neither. One variant was obtained that displayed insignificant uninduced killing and complete induced killing.

(e) aTc-inducible killing transfer curve for the ile-2 single gRNA, initial 2-gRNA, and optimized 2-gRNA kill switches after 1.5 h of induction in LB. Points represent experimental data while lines represent the fitted curves.

For all kill switch assays, exponential phase cells for each strain were induced with the specified aTc concentrations or 0 and 100 ng/mL aTc for 1.5 h or the specified amount of time. Values and error bars are the average and standard deviation of biological triplicate, respectively. Source data are provided as a Source Data file.

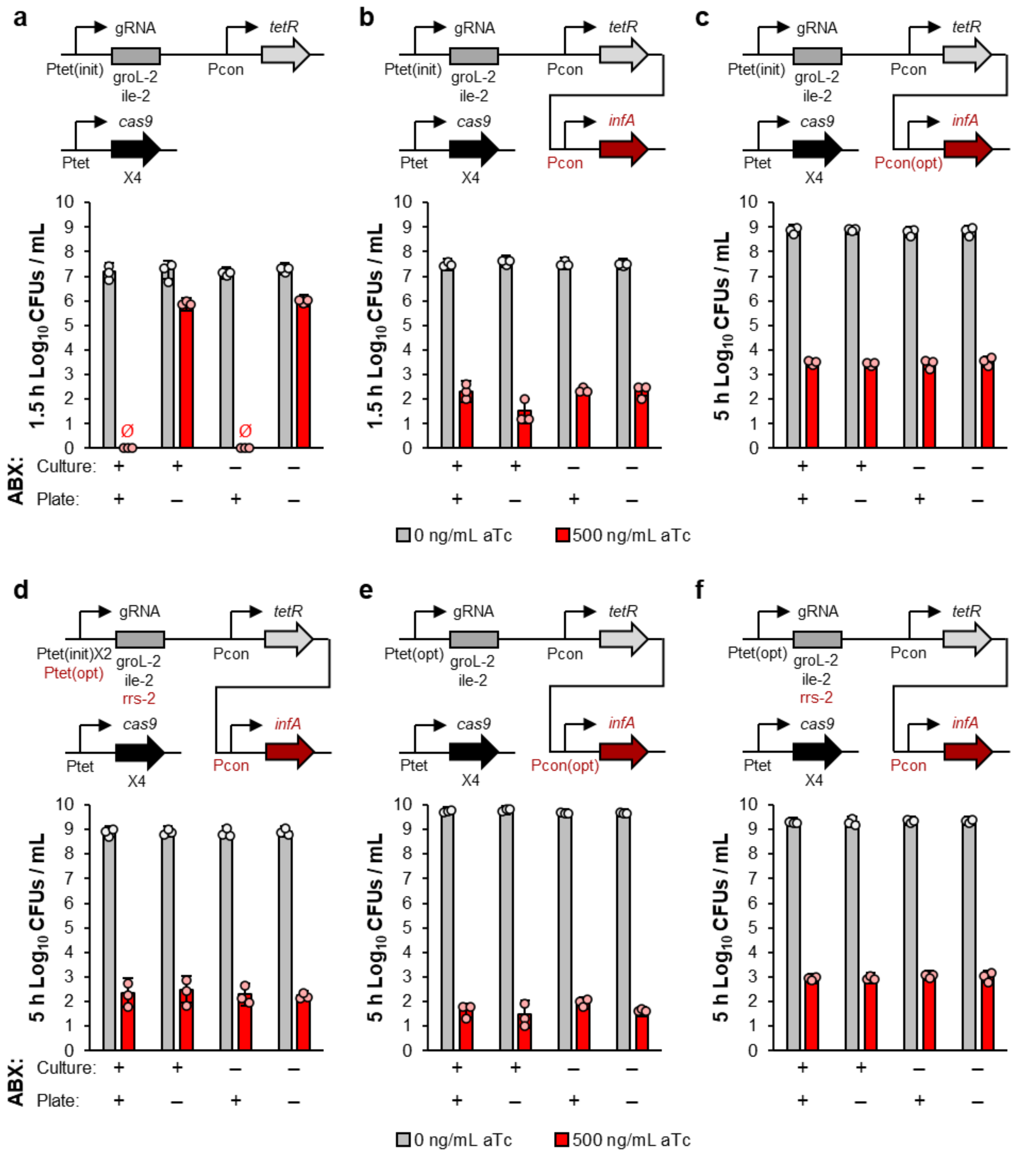

Supplementary Fig. 2 Multi-method approach to developing an optimized antibiotic-independent CRISPRks, related to Fig. 2

(a-f) System schematics and $\log_{10}$ CFU values for the following kill switch strains:

(a) initial 2-gRNA circuit, which has four genome-integrated P_{tet}-*cas9* expression cassettes (X4) and unoptimized groL-2 and ile-2 P_{tet}-gRNA expression cassettes.

(b) ABX-independent initial 2-gRNA circuit, which has four genome-integrated P_{tet}-*cas9* expression cassettes (X4), unoptimized groL-2 and ile-2 P_{tet}-gRNA expression cassettes, and an unoptimized constitutive *infA* expression cassette to complement a genomic *infA* knockout.

(c) ABX-independent *infA* optimized initial 2-gRNA circuit, which has four genome-integrated P_{tet}-*cas9* expression cassettes (X4), unoptimized groL-2 and ile-2 P_{tet}-gRNA expression cassettes, and an optimized constitutive *infA* expression cassette to complement a genomic *infA* knockout.
(d) ABX-independent single gRNA optimized initial 2-gRNA circuit, which has four genome-integrated P_{tet}-*cas9* expression cassettes (X4), unoptimized groL-2 and ile-2 P_{tet}-gRNA expression cassettes and an optimized rrs-2 P_{tet}-gRNA expression cassette, and an unoptimized constitutive *infA* expression cassette to complement a genomic *infA* knockout.
(e) ABX-independent *infA* optimized, optimized 2-gRNA circuit, which has four genome-integrated P_{tet}-*cas9* expression cassettes (X4), unoptimized groL-2 and ile-2 P_{tet}-gRNA expression cassettes, and an optimized constitutive *infA* expression cassette to complement a genomic *infA* knockout.
(f) ABX-independent gRNA optimized, optimized 2-gRNA circuit, which has four genome-integrated P_{tet}-*cas9* expression cassettes (X4), optimized groL-2, ile-2, and rrs-2 P_{tet}-gRNA expression cassettes, and an unoptimized constitutive *infA* expression cassette to complement a genomic *infA* knockout.
Exponential phase cells for each strain were induced with 0 and 500 ng/mL aTc for 1.5 h (A and B) or 5 h (C-F) in LB with and without spectinomycin. CFUs were determined by plating onto LB agar with and without spectinomycin. Key differences between the circuits are in red. *gRNA*, *tetR*, and *infA* cassettes are located on the same plasmid (connected lines), while cas9 is located exclusively in the genome. Values and error bars are the average and standard deviation of biological triplicate, respectively. Source data are provided as a Source Data file.

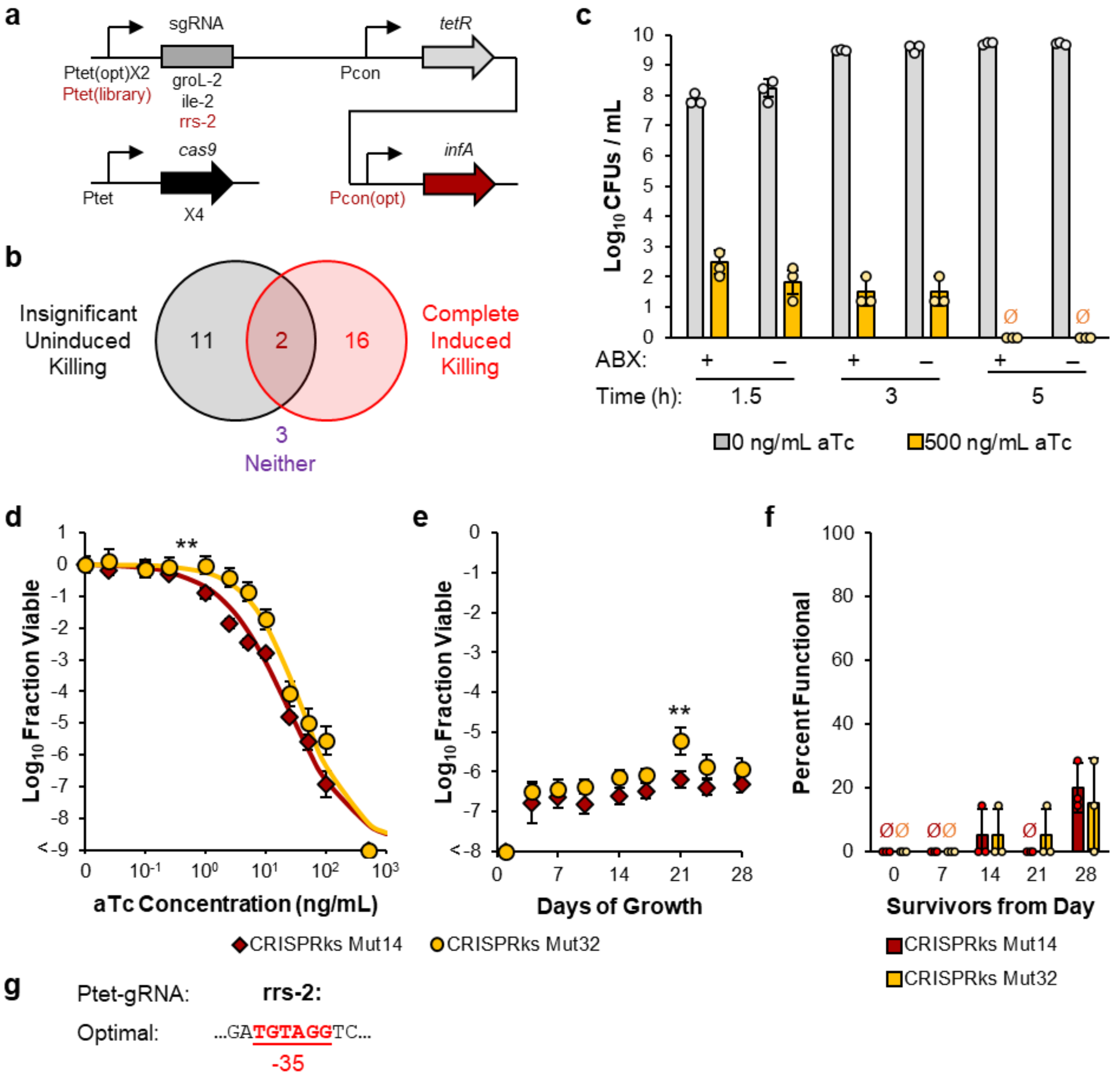

Supplementary Fig. 3 Selecting a final optimized antibiotic-independent CRISPRks, related to Fig. 2

(a and b) Library schematic and screening results for incorporating a rrs-2 gRNA expression cassette into the optimized antibiotic (ABX)-independent 2-gRNA kill switch. (b) Number of library variants that demonstrated insignificant killing in response to 0 ng/mL aTc, complete killing in response 500 ng/mL aTc, both, or neither. Two kill switches were obtained that displayed insignificant uninduced killing and complete induced killing (Mut14 and Mut32).

(c) Log_{10} CFU values for the Mut32 CRISPRks, which has four genome-integrated P_{tet}-*cas9* expression cassettes, optimized groL-2, ile-2, and rrs-2 P_{tet}-gRNA expression cassettes, and an optimized constitutive *infA* expression cassette to complement a genomic *infA* knockout. Exponential phase cells for each strain were induced with 0 and 500 ng/mL aTc in LB with and without spectinomycin. CFUs were determined by plating onto LB agar with or without spectinomycin.

(d) aTc-inducible killing transfer curves for the Mut14 and Mut32 CRISPRks strains after 3 h of induction in LB without antibiotics. Points represent experimental data while lines represent the fitted curves.
(e) Long-term stability assessment of the Mut14 and Mut32 CRISPRks strains. Each day for 28 days, three replicates of each strain were diluted 250X into fresh LB without antibiotics and grown for 24 h. Every 3-4 days, exponential phase cells were induced with 0 and 500 ng/mL aTc for 3 h and plated on LB agar without antibiotics for CFU quantification.
(f) Percent of Mut14 and Mut32 CRISPRks cells that survived aTc induction with non-functional kill switches. To assay for kill switch functionality, exponential phase cells were induced with 0 and 500 ng/mL aTc for 3 h and the absorbance at 600nm quantified. Induced to uninduced absorbance ratios within three standard deviations of the no gRNA control strain were deemed non-functional. 24 colonies (8 from each replicate) at selected timepoints from Supplementary Fig. 3e were tested for functionality.
(g) Mut14 was selected for further characterization due to its superior aTc response time, aTc sensitivity, and efficiency after long-term growth.
Fraction viable values of <-8 or <-9 had no colonies obtained from cultures receiving aTc. Values and error bars are the average and standard deviation of biological triplicate, respectively. Statistical comparisons were performed using two-tailed mixed model ANOVA (**, $P < 0.01$). Source data with p-values are provided as a Source Data file.

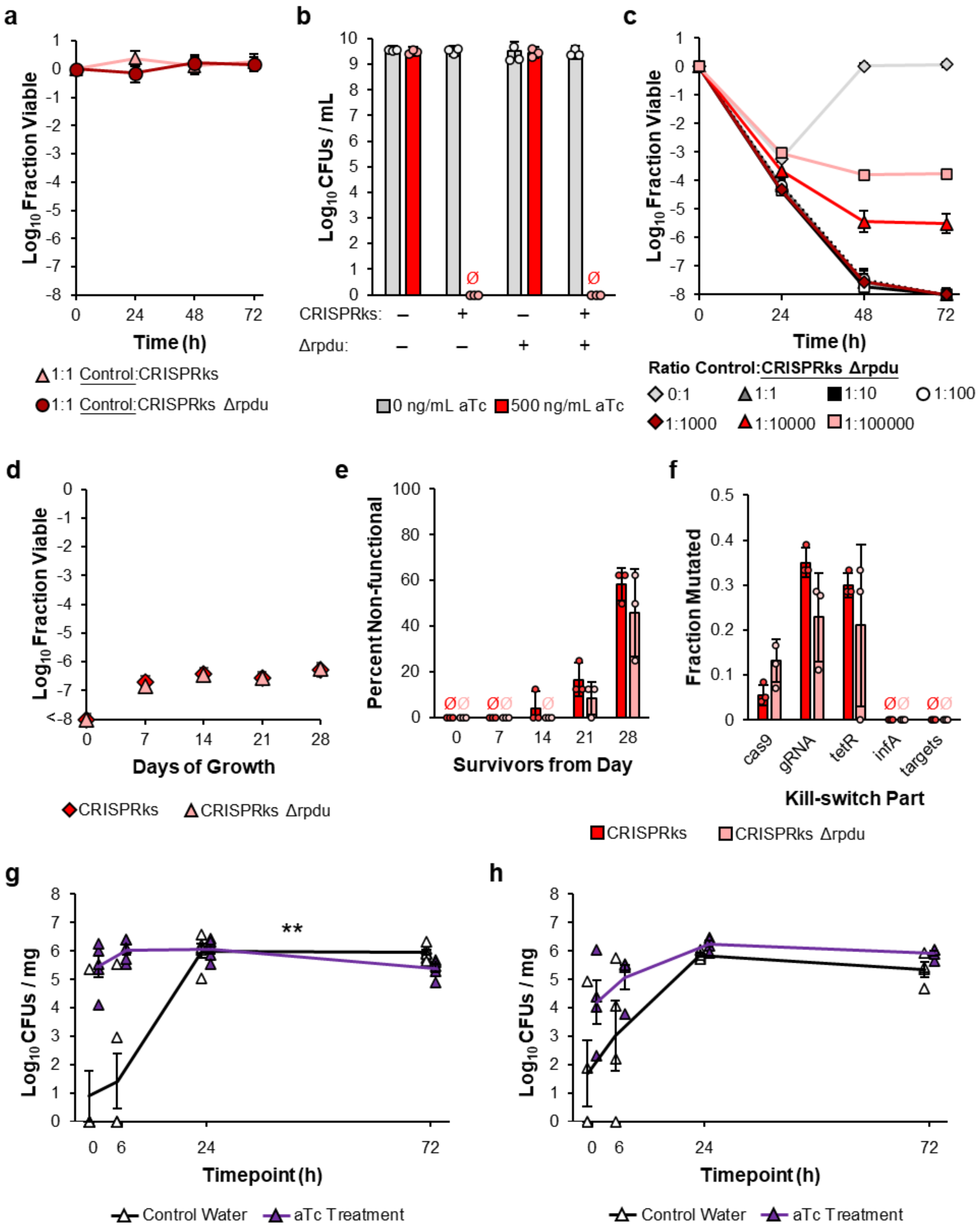

Supplementary Fig. 4 Knocking out key components of the SOS response does not impact the long-term stability of the CRISPRks and minimally affects the growth and aTc-response of no gRNA control strains, related to Fig. 3

(a) Log_{10} Fraction Viable of the no gRNA control strain with and without the Δrpdu knockouts when incubated in a 1:1 ratio mixture with the CRISPRks or CRISPRks Δrpdu strains, respectively. Cells were cultured anaerobically at 37°C without shaking in M9+0.4% glucose and induced with 0 or 500 ng/mL aTc. Values and error bars are the average and standard deviation of biological triplicates, respectively.
(b) Log_{10} CFU values for the no gRNA control (– CRISPRks) and CRISPRks strain with and without knockouts (Δrpdu). Exponential phase cells for each strain were induced with 0 and 500 ng/mL aTc for 3 h in LB without antibiotics. CFUs were determined by plating onto LB agar without antibiotics. Values and error bars are the average and standard deviation of biological triplicates, respectively.
(c) Log_{10} Fraction Viable of CRISPRks Δrpdu when incubated with the no gRNA control strain at different ratios. Cells were cultured anaerobically at 37°C without shaking in M9+0.4% glucose and induced with 0 or 500 ng/mL aTc. Values and error bars are the average and standard deviation of biological triplicates, respectively.
(d) Long-term stability assessment of CRISPRks and CRISPRks Δrpdu. Each day for 28 days, three replicates of each strain were diluted 250X into fresh LB without antibiotics and grown for 24 h. Every 7 days, exponential phase cells were induced with 0 and 500 ng/mL aTc for 3 h and plated on LB agar without antibiotics for CFU quantification. Fraction viable values of <-8 had no colonies obtained from cultures receiving aTc.
(e) Percent of CRISPRks and CRISPRks Δrpdu cells that survived aTc induction with non-functional kill switches. To assay for kill switch functionality, exponential phase cells were induced with 0 and 500 ng/mL aTc for 3 h and the absorbance at 600nm quantified. Induced to uninduced absorbance ratios within three standard deviations of the no gRNA control strain were deemed non-functional. 24 colonies (8 from each replicate) of each strain at each timepoint in Supplementary Fig. 4d were tested for functionality. Values and error bars are the average and standard deviation, respectively.
(f) Sequencing results from 24 CRISPRks and 24 CRISPRks Δrpdu survivors (8 from each replicate) from Supplementary Fig. 4d with non-functional kill switches. The fraction mutated is the fraction of total sequenced cassettes that contained a mutation. Values and error bars are the average and standard deviation, respectively.
(g and h) Log_{10} CFUs/mg feces of the no gRNA control strain (g) without or (h) with the Δrpdu knockouts from mice receiving control water or aTc treatment water. Points are the average of two technical replicates. Lines and error bars are the average and standard error from 6 (g) or 4 (h) mice across two cages, respectively. Statistical comparisons were performed using two-tailed mixed model ANOVA (**, $P < 0.01$). Source data with p-values are provided as a Source Data file.

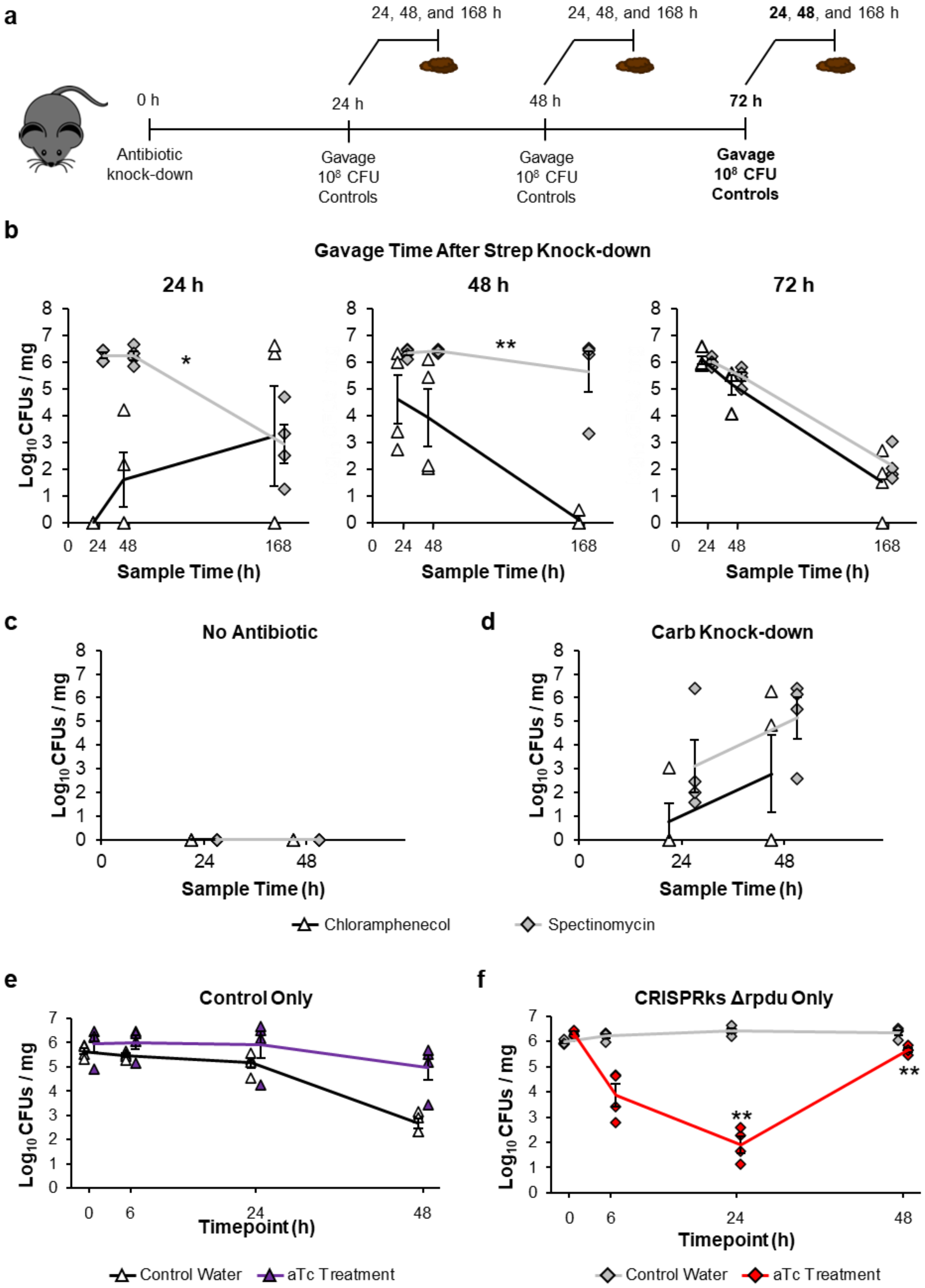
a
24, 48, and 168 h
24, 48, and 168 h
24, 48, and 168 h
0 h
24 h
48 h
72 h
Antibiotic knock-down
Gavage 10⁸ CFU Controls
Gavage 10⁸ CFU Controls
Gavage 10⁸ CFU Controls
b
Gavage Time After Strep Knock-down
24 h
48 h
72 h
Log10 CFUs / mg
Sample Time (h)
c
No Antibiotic
d
Carb Knock-down
Chloramphenecol
Spectinomycin
e
Control Only
Timepoint (h)
Control Water
aTc Treatment
f
CRISPRks Δrpdu Only
Control Water
aTc Treatment

Supplementary Fig. 5 Optimization of the antibiotic knockdown and EcN gavage protocol as well as *in vivo* results for single gavage of the no gRNA control and CRISPRks Δrpdu strains, related to Fig. 3

(a) Schematic for testing different antibiotic knockdown and EcN gavage protocols. 24 (b only), 48 (b only), and 72 h after antibiotic treatment, 10^8 CFUs of chloramphenicol- or spectinomycin-resistant no gRNA control strains were gavaged into C57BL/6 mice. Fecal samples were collected 24, 48, and 168 (b only) h after gavage for CFU quantification.

(b-d) Log_{10} CFUs/mg feces of the chloramphenicol- and spectinomycin-resistant no gRNA control strains from mice receiving (b) a streptomycin knockdown, (c) no antibiotics, or (d) a carbenicillin knockdown.

(e and f) Log_{10} CFUs/mg feces of the (e) no gRNA control or (f) CRISPRks Δrpdu strain from mice receiving each strain alone and control water or aTc treatment water.

Points are the average of two technical replicates. Lines and error bars are the average and standard error from 4 mice across two cages, respectively. Statistical comparisons were performed using two-tailed mixed model ANOVA (f) or two-tailed mixed model ANOVA with Sidak's multiple comparisons (b) (*, $P < 0.05$; **, $P < 0.01$). Source data with p-values are provided as a Source Data file.

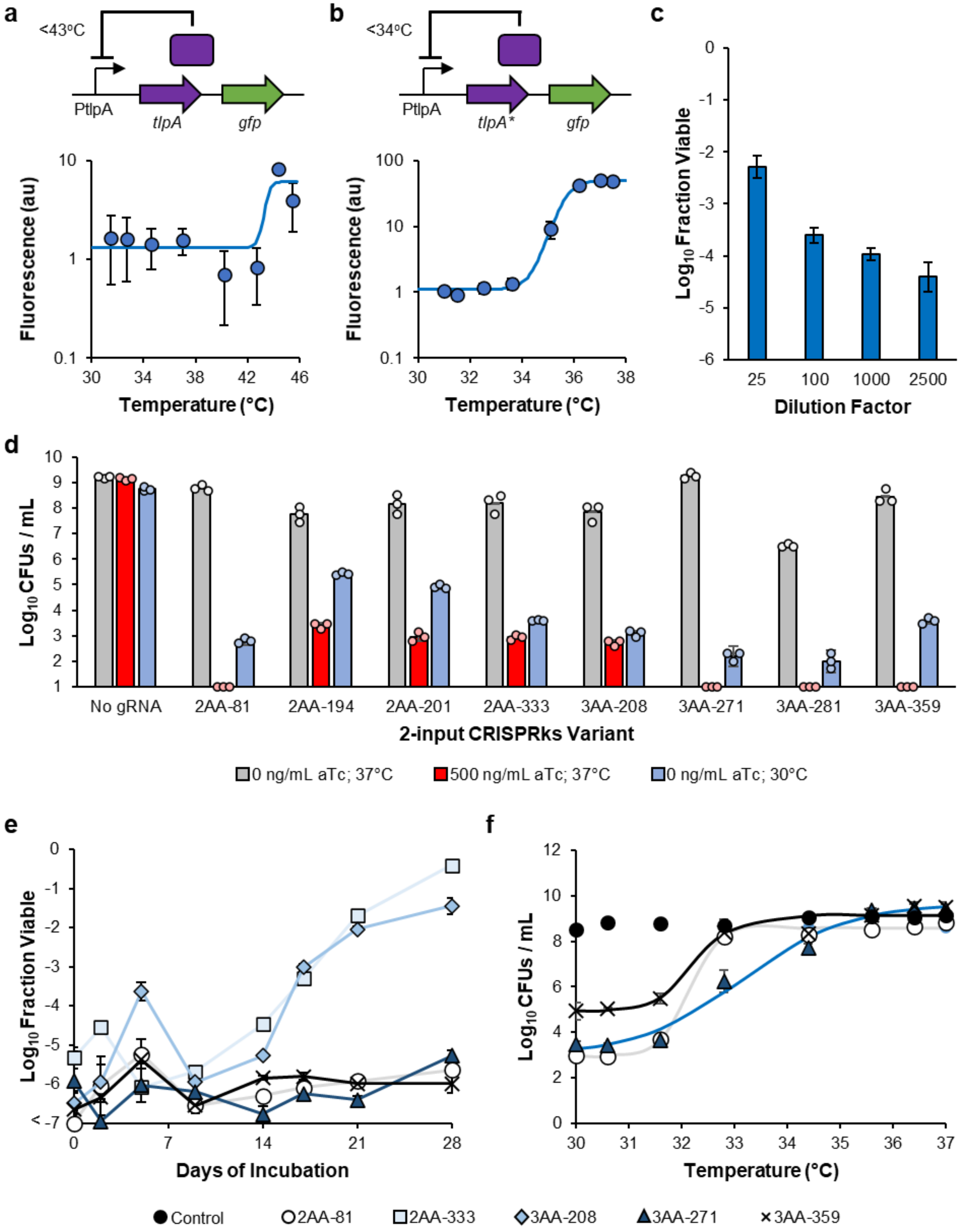

Supplementary Fig. 6 Development and selection of an optimized 2-input CRISPRks Δrpdu kill switch, related to Fig. 4

(a and b) Temperature sensing schematics and transfer curves for the (a) wild-type TlpA and (b) modified TlpA* temperature sensors. The *tlpA*/*tlpA** temperature sensors and *gfpmut3* are

expressed in an operon from the P_{tlpA} promoter. At low temperatures, TlpA/TlpA* proteins form homodimers and repress transcription from the P_{tlpA} promoter. TlpA was previously engineered to have a half-maximal expression at ~36°C in *E. coli* NEB10β, resulting in TlpA*. EcN with the temperature sensing plasmids was grown at (a) 31.5, 32.7, 34.6, 37, 40.2, 42.7, 44.4, and 45.5°C or (b) 31, 31.5, 32.5, 33.6, 35.1, 36.2, 37, and 37.5°C for 24 h, and the fluorescence was quantified by flow cytometry. Points represent experimental data while lines represent the fitted curves.
(c) Log_{10} Fraction Viable for the initial 2-input CRISPRks when incubated at different fold dilutions. Exponential phase cells were diluted 25, 100, 1000, and 2500X into fresh LB without antibiotics and incubated for 3 h at 30 and 37°C. Cultures were then plated on LB agar without antibiotics and incubated overnight at 37°C for CFU quantification.
(d) Log_{10} CFUs for the no gRNA control, initial 2-input CRISPRks, and optimized 2-input CRISPRks. Exponential phase cells for each strain were induced with 0 and 500 ng/mL aTc at 37°C for 3 h. Cultures were then plated on LB agar without antibiotics and incubated overnight at 37°C (both 0 and 500 n/mL aTc cultures) or for seven days at 30°C (0 ng/mL cultures) for CFU quantification. The 2AA-81, 2AA-333, 3AA-208, 3AA-271, and 3AA-359 variants were selected for further characterization.
(e) Long-term stability of the five 2-input CRISPRks strain selected from Supplementary Fig. 6d. Each day for 28 days, three replicates of each strain were diluted 250X into fresh LB without antibiotics and grown for 24 h. Every 3-4 days, exponential phase cells were plated on LB agar without antibiotics and incubated overnight at 37°C and for 7 days at 30°C for CFU quantification. Fraction viable values of <-7 had no colonies obtained from cultures receiving aTc. The 2AA-81, 3AA-271, and 3AA-359 variants were selected for further characterization.
(f) Cell death transfer curve with respect to temperature for the no gRNA control and the three 2-input CRISPRks variants selected from Supplementary Fig. 6e. Exponential phase cells were incubated in a thermocycler for 5 h at a range of temperatures: 30, 30.6, 31.6, 32.8, 34.4, 35.6, 36.4, and 37°C. Points represent experimental data while lines represent the fitted curves. Control values were not fit due to a lack of response. The 2AA-81 2-input CRISPRks variant was selected for further characterization.
Values and error bars are the average and standard deviation of biological triplicate, respectively. Source data are provided as a Source Data file.

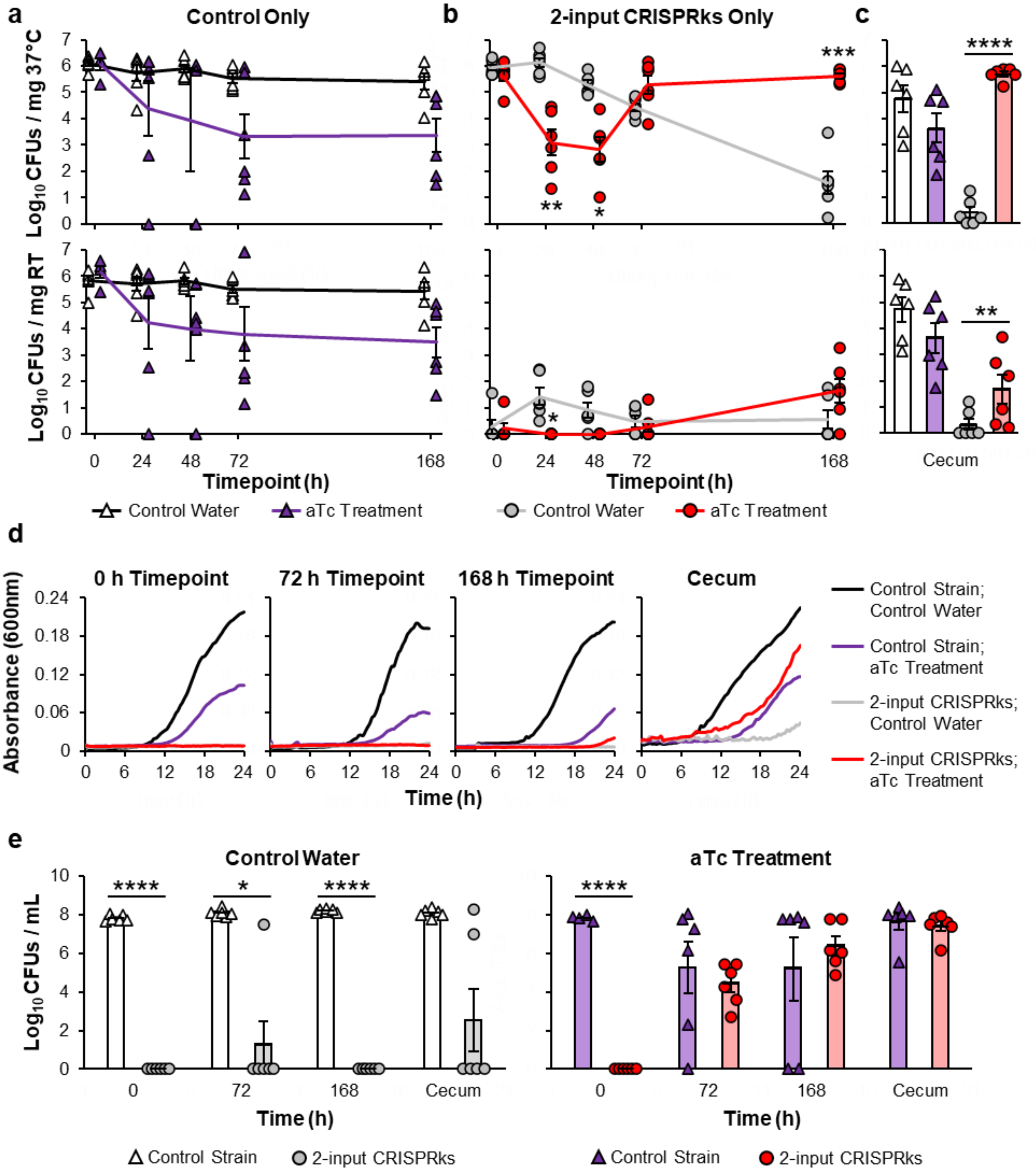

Supplementary Fig. 7 Temperature and aTc response of the 2-input CRISPRks in the absence of intra-niche competition, related to Fig. 5

(a-c) Log_{10} CFUs/mg feces (a and b) or cecal contents (c) of no gRNA control (a and c) or 2-input CRISPRks (b and c) cells from mice receiving the no gRNA control strain alone or the 2-input CRISPRks strain alone. CFUs were quantified at 37°C (top) or RT (bottom). Points are the average of two technical replicates. Lines and error bars are the average and standard error from 6 mice across two cages, respectively.

(d) RT growth assays for no gRNA control and 2-input CRISPRks cells obtained from fecal or cecal samples at different timepoints in Supplementary Figs. 7a-7c.

(e) CFUs of no gRNA control and 2-input CRISPRks cells from Supplementary Fig. 7d following 24 h of RT growth. Points are the average of two technical replicates. Values and error bars are the average and standard error from 6 mice across two cages, respectively.
Statistical comparisons were performed using two-tailed mixed model ANOVA with Sidak's multiple comparisons, (*, $P < 0.05$; **, $P < 0.01$; ***, $P < 0.001$, ****, $P < 0.0001$). Source data with p-values are provided as a Source Data file.

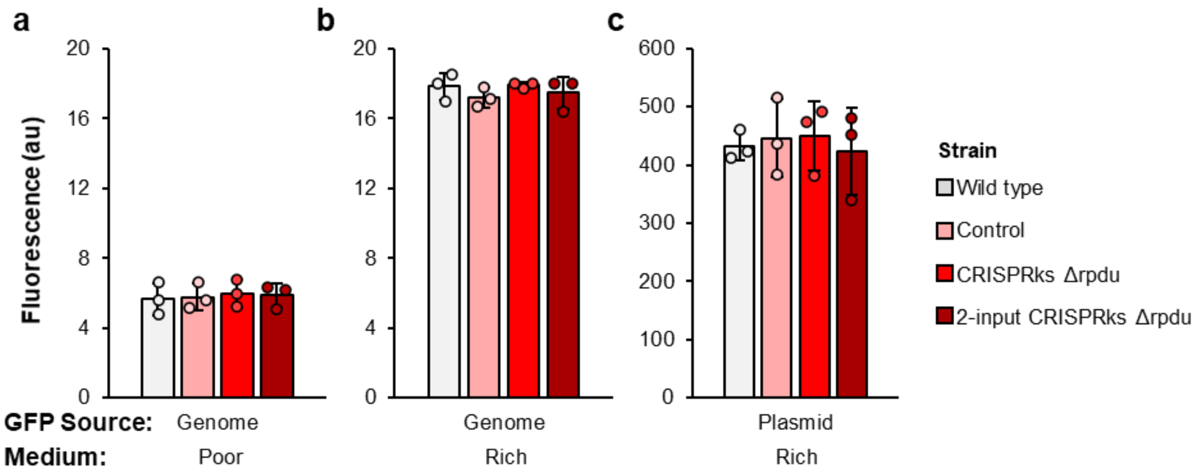

Supplementary Fig. 8 The CRISPRks Δrpdu and 2-input CRISPRks Δrpdu strains can express heterologous proteins from the genome and on plasmids at levels equivalent to wild-type EcN

(a-c) Fluorescence of constitutively expressed GFP in wild-type, control, CRISPRks Δrpdu, and 2-input CRISPRks Δrpdu EcN strains. GFP was expressed from (a and b) a genome-integrated cassette, or (c) a medium-copy plasmid-based cassette. Stationary phase cultures were diluted 100X into (a) M9+0.4% glucose (poor) or (b and c) LB medium (rich) and incubated for 6 h before fluorescence quantification. Values and error bars are the average and standard deviation of biological triplicate, respectively. Source data are provided as a Source Data file.

Supplementary Table 1 Fitted Hill equation parameters, related to Figs. 2, 4, and Methods. The Hill equation, described in the Methods, was fit to each set of normalized fluorescence (au) values by minimizing the root mean square error (RMSE). Fmax and Fmin represent the fitted maximum and minimum normalized fluorescence (au) values, CFUs/mL, or Fraction Viable, respectively. The half-maximal constant (K_A) is in mM of the respective ligand or ^oC. The fitted Hill coefficient (n) and RMSE are also reported. FV, Fraction Viable. S before figure numbers, Supplementary.

Figure	Strain	Plasmid	Fmax	Fmin	n	K_A	RMSE
2d and S3d	sAGR514	pAGR401	1 FV	$10^{-9.00}$ FV	0.76	21.0 ng/mL	1.08
4e and S6f	sAGR596	pAGR510	$10^{8.86}$ CFUs/mL	$10^{2.91}$ CFUs/mL	123	32.1°C	0.06
S1e	sAGR039	pAGR318	$10^{7.79}$ CFUs/mL	$10^{3.07}$ CFUs/mL	8.33	81.9 ng/mL	0.28
S1e	sAGR514	pAGR378	$10^{7.19}$ CFUs/mL	0 CFUs/mL	4.27	3.09 ng/mL	0.02
S1e	sAGR514	pAGR384	$10^{8.31}$ CFUs/mL	0 CFUs/mL	1.37	18.7 ng/mL	0.45
S3d	sAGR514	pAGR402	1 FV	$10^{-8.81}$ FV	0.98	37.5 ng/mL	1.47
S6a	sAGR039	pAGR414	6.10 au	1.31 au	185	43.4°C	11.0
S6b	sAGR039	pAGR415	50.0 au	1.10 au	109	35.6°C	2.30
S6f	sAGR596	pAGR513	$10^{9.67}$ CFUs/mL	$10^{3.02}$ CFUs/mL	33.4	33.2°C	0.28
S6f	sAGR596	pAGR514	$10^{9.14}$ CFUs/mL	$10^{4.93}$ CFUs/mL	86.3	32.3°C	0.32

Supplementary Table 2 gRNA and oligo sequences used for knockouts, related to Methods.

Gene(s)	gRNA Sequence	60bp Oligo Sequence
infA	gtgatatgtatgaagtactt	cggtgcttgttttcaccacaagaatgaatgtgtctcgatgtagatatactgcacaacttg
recA	ctactgcgccagaacgtgcc	ttcgctgtcatctacagagaaatccggcgttttctcaatctggcccagtgctgccgccaa
polB	catgccttccaccagtccga	gtggcgcaggcaggttttatcttaacccgacttatgaccgggcaacttgggctattttga
dinB	cggcgtcggcaaagtctcgg	tcataatcccagcaccagttgtctttccatgtccatatccacatgaatgattttacgcat
umuDC	tctccccgtggatgaagtct	cgttgtataacatggtgaaagcatggcacgatcgctaaatagcggaaaagtcacaatttc

Supplementary Table 3 gRNA and homologous arm sequences used for insertions, related to Methods.

Insert	Insertion Region	gRNA Sequence	Upstream Homologous Arm	Downstream Homologous Arm
p19-*gfpmut3*	*torST*	tgaccagtaccc tggtggga	tgttgctacctgagtacgaacacccggatcttcaatg cgtatttgccgacgttgcagaattttcaccgcattattg agctgcttttccagcgttggcgcatttttctggatctgttc cagccccagattcatcaccatttgctgcacccgcag ggcgctaaggcgcagttcattcatctggttaacatact caagatcgatatcaatcagccgatcgagtgcactttc tgcggcctgacgctgatgttgttcgatcaaatcgtaaa tcccggcctgggtcgctccggcggaggttgccgcatt attcgcctgaccttgcgccaggcgtgcgatttcatcgg cggcggcgactatctgctgactgagttgctgttgttgct ggcgaagttgcaaacgctgccccaccagttcccctt gctgacgtaacgaacgggagatctcctgctcctgttg ttcaatagcggtggtgtcaaaaccttgttcccgtaacg cttgcagcaacgcattaatcttcaggctttgtgcggtg	ggtcggtgcactttaggtgaaaaaggttgagtcgcaa agcagaatgcatctagcataaagccttattattgatga ggctatcatgcgcgtactgctatttttacttctttccctttttc atgttatcggcattttcggctgataacctgttgcgctgg catgatgcgcagcattacacggtgcaagcctctacg ccgcttaaagccaaacgcgcatggaaactgtgcgc gctttatcccagcctgaaagattcatactggttatcgtt gaactatggtatgcaggaggctgctcgccgctacgg tgtggatttaaaagtgctggaggcaggcggctacag ccagttggctacgcagcaagcacaaatcgaccagt gtaaacagtggggcgcagaggccatttgctcggta gtagcacgacgtcatttcccgatctgcaaaagcaggt agcaagtctgccggtgatcgaactggtgaatgctatt

			agcattcgcccctgcgcctgccacatcttttcgttatcggcactggtcaggttctgcgcggcgaaaagttcccaggcgctggc	gatgctccccacgtgaaaagccgcgttggtgtgccctggtttcagatgggctatcaaccaggacgatatctgg
Ptet-*cas9*	*lacZ*	gatagttttcttgcggcccc	aggatgagtgcacagccagaacccagccagaaaacaaactgattattgatggtgaacatgatgccgacaatcgaggcacacagcgcccagccaacacagccaaacatccgcgcgcgaccaaattcgaaattactgcgacggctgactttctcgataaatgcctctactgctggcgcaccggcgttaaaacaaaagccaagataaataccaccaacaatcgatcctactaaaatgttgtattgtaacagtggtccgaagataaaaataaagaacggcgcaaacatcactaacatgccggtaataatccacagcaggtatttgcgcagcccgagtttgtcagaaagcagaccaaacagcggttggaataatagcgagaacagagaaatagcggcaaaaataatacccgtatcacttttgctgatatggttgatgtcatgtagccaaatcgggaaaaacgggaagtaggcccccatgataaaaaaagtaaaagaaaaagaataaaccgaacatccaaaagtttgtgtttttaaatagtacataatggatttccttacgcgaaatacgggcagacatagcct	agctgtatcctgtgtgaaattgttatccgctcataatttcacacaacatacgagccggacacatagagtgtaaagactggggtgcctaatgagtgagctgactcacattaattgtgttgcgctcactgcccgctttccagtcgggaaacttgtcgtgccagctgcattaatgaatcggccaacgcgcggggagaggcggtttgcgtattgggcggaagggtggtttttcttttcaccagtgagacgggcaacagctgattgcccttcaccgcctggccctgagagagttgcagcaagcggtccacgctggtttgccccagcaggcgaaaatcctgtttgatggtggttaacggcgggatataacacgagctgtcttcggtatcgtcgtatcccactaccgagatatccgcaccaacgcgcagcccggactcggtaatggcgcgcattgcgcccagtgccatctgatcgttggcaaccagcatcgcagtgggaacgatgccctcattagcatttgcatggtttgttgaaaaccggacatggcactccagtcgccttcccgttctgttatcggctgaatttgattgcgtgtgagatatttatgccagccc
Ptet-*cas9*	*agaI*/*rsmI*	agcgctacaacgcctttacc	agcgaatatttattggccgtgatccgtcgcaaaccggatgccgtgatttgcctggcgaccggagccacgccattactgacgtatcattatctggtagaaaaaaatccaccagcagcaggttgatgtcagccatctcaccttcgtgaagctcgacgaatgggtggatctgccattaacgatgcctggcacctgcgaaactttcctacaacagcatatcgtgcagccgctggggctgcgtgaagaccagctcatcagctttcgctccgaagagataaatgagacagagtgcgaacgggtaacgaacctgattgcgcgcaaaggcggtctggatttatgcgttctcggattggggaaaaacggtcatcttgggctgaacgaaccgggagaaagcctgcaaccagcctgccatatcagtcaacttgatgccagaacacagcaacatgagatgttaaaaaccgcggaccgtcccgtgactcgtgggatcaccttaggcctgaaggatattctcaatgcccgcgaagttttgttactggtgactggcgaaggaaaacaggatgcgacagaacgttttctcacggctaaagtctccaccgctatcccggcttcatttttatggctgcataaccatttcacatgcgtgattgatgaaatgtgtcgtcgttaa	ctgcgacgcctgtaatcactctccctgctgctccagcgcatacttatacagcgcattttcttcacgccgtgaatttctgcggccagcgccgccgctttttcagcggcagttctgcctgtagcagcgccagcgtgcgcagggcatcagcaggcaagtcattttcctgcgctttatggccttcgacaatcagcaccatttcgcctttgcgacggttttcatcttcttttacccacgccagcagctcgccaacgggcgcgcccgtgaatggtttcccaggttttggttagctcacgcgccagaaccacgtaacgggattcgcctaataccgcaacgatatcttccaggctatctaacagacggtgggtagattcataaaaatcagcgtgcgcggctccgcttcaatagcttttagcgcatcacggcggcccttttgatttggcaggtaaaaagccttcataacaaaaacgatcggatggtaaacccgcggcgcttaacgcagtgatagcagcacacggccctggtagcggcaccacgcgaatccccgcttcacgacaggtacgcaccagatggtagccaggatcgttaattagcggcgttccggcatcggaaaccagcgcaatgttt
Ptet-*cas9*	*rhtBC*	gacgcgtcatcagagtaagt	aggatgaagactccgtaaacgtttcccccgcgagtctaatgtatgttgatgttattcctcaccgtcgccatggtgcacattgtggcgcttatgagccccggtcccgatttcttttttgtctctcagaccgctgttagtcgttcccgtaaagaagcgatgatgggcgtgctgggcattacctgcggcgtaatggtttgggctgggattgcgttgcttggcctacatttgattatcgaaaaaaatggcctggctgcatacgctgattatggtgggcggtggcctgtatctctgctggatgggttaccagatgctacgtggtgcactgaaaaaagaggttgtttctgcccctgcgccacaagtcgaactggcgaaaagcgggcgcagttttcttaagggcttactgaccaatctcgcgaaccctaaagcgattatctactttggttcggtgttctcgctgtttgtcggtgataatgtcggcaccaccgaacgctggggcattttcgcgttgattattgtcgaaacactggcgtggtttaccgtcgttgccagcctgtttgctctgccgcaaatgcgccgtggttatcaacgtctggcgaagtggattgatggttttgccggggcgttatttgccggatttggcattcatttgattatttcgcgttaatg	tattttcacgcatgcctcgccgatgctaacagcgctcccaccagcataaataacgagccgaaaatcttattcagcgccttcatctgctttggtcctttaatccatagagcaatccgttgagcaagggtggcgtaaccgatcatcacaataatatcgaccacaatagtggtgacgccgagcacgatatactgcatcagttgtggctgctgcggcatgatgaattgcggaaatagcgccgccagaaacacacaatacttttgggattggtgagattcacaaaaactgcgcgctggaacaaatgtcgacgcgattgagtggaggccagcgatttaagatcaattgcgccagcggcgcgccattgctgtattcccagccaaatcaagtaagctgcgcctgcccacttcaacacttcaaacgcaatcactgagcgggaaaatagcgtccccaaccccacgccaaccagcacaatatgaatcgccagtccggtctgaagcccagcaatagacgccaccgcgccgcgataaccgtggttgagcgaggtggtcatggtgttgattgcgccagagcctggcgacaggcttaaaatgatcgatgtcagcaggtaggcaaaccaccattctaaggtcatgatgaactcccggtgt
Ptet-*cas9*	*exo*/*cea*	ctggataacgtaaatgattg	cattgttcttcgaaggctgttgggatactggccgggatatttatcctgtagcatctccagcagagttgttggtgtcagagccggcctctctttcaacagaggaacaagcatgctgtcccacatagcttccagaggaagctttgcgtgtgcgccagtgccgaacactgttgttttcccactctcctttttcgatccgacgaccagaatggactgagataccagccttcatggccgagatatgcggtcggtcaggtggtatttgaaaccagactgaagcccacggtcgtcatcgggacgcaggccgccgccgtgcatggtatcagtgagcaggcactctgcggagcaccatcatggactgatgtggtgcggcaactgcgtcatgcaatcggggaccgaccagt	caataaatagcaatcccccagatacctaatgtagttccagcaagcacggccgggcagcttgttctgcctgcgttttcttcaattgagcagtagaccatttagctgtggcatgaatggctgcagaactttcactgttgctacctccagttccaccaccgctgccagagccactcccgtctggattatcattcaaaagagtaatgattacctgccccttatcatcataaggaacaccatctttatagtacgctacagctgtttccattataaaatcctctttgacattaaaaacaatcagttaaaaataagtactgcatatataattactggttttatatacagcataaaaattacgccgctgcgttttccctgtcaaccctgtggattttcatttttgtgaaaacgatcaaaaaaacaactact

	aattatctttaatgcccggttcgacatccgcattctgaaaaagactgctgccgcacatagcgatccggctgactggctggaagaactgacggtatattgtgtgatggagctggctgcaggatattatggagcctccaaccgctatggcactatttcactggcctgtgctgccagccagaccggactgaactgggaagggcaggcacactcagcgatcgctgacgcacggatgacggcaggggtggtaaacgctattgctgcatatcatctggaactgctgcaggaacaggcacggctgaaaacctgactgcctggcctgtata	cacaattcgacagtcccgccagataccgcaaaaccggccagacgttaccgttttccgggaccatgatatgagccccgttggggagggtatggagttgctgaaaatgacggtcagattgagttcaccgttttattgttacaggaggccagggcttgtctgctaccggtccggacgagagggataccgggaatttgaatccggttaactgagccggacatacggtaaataaggaatgcaggcagaacgggagtcactgcaggatgagaccggcactacagcagaaaaacaggtgattctggtacggctcagccactaaacacagttggaaactgatgataagatagtcagcggttatataactcactgagtaaaaagatataaaggtaaggagacactcatgagtcg

Supplementary Table 4 Sequencing primers, related to Methods.

Target Region	Sequence
Ptet-*cas9* plasmid forward	GCAGTTTCATTTGATGCTCGATG
Ptet-*cas9* plasmid reverse	GATACTGTGGCGGTCTGTATTTCC
lacZ Ptet-*cas9* integration forward	GTTGATGTCATGTAGCCAAATCG
agaI/rsmI Ptet-*cas9* integration forward	GTTTTCTCACGGCTAAAGTCTCC
rhtBC Ptet-*cas9* integration forward	GAAGTGGATTGATGGTTTTGCC
exo/cea Ptet-*cas9* integration forward	CGCTATGGCACTATTTCACTGG
All cas9 Ptet-*cas9* integrations reverse	ATCCGCTTTATCAGTAGAATCTACC
gRNA Plasmid spot 1	GCGCATTAGAGCTGCTTAATGAGG
gRNA Plasmid spot 2	CCTGCAGCACTGTCCTCTGCTCCTGATACC
gRNA Plasmid spot 3	CTTCATCCGTTTCCACGG
gRNA Plasmid spot 4	GGGCAAATATTATACGCAAGGC
groL-2 target site forward	GGCAATTGTTGAAGCGTAATCC
groL-2 target site reverse	CGTCGGAGTTAGCAGAGATGG
ileTUV-2 target sites forward	CAGAATGCCACGGTGAATACG
ileTUV-2 target sites reverse	GAGCAAAACTTCGCAGTGAACC

Supplementary Table 5 Plasmids used in this work, related to Methods.

Plasmid Name	Genetic Parts	Origin	Antibiotic Resistance	Source
**Recombineering**				
pgRNA	Constitutive gRNA	pBR322	Chloramphenicol	1
pMP11	Pcon-*cas9* + pBAD-λ Red genes + Ptet-gRNA-pBR322ori	oriR101	Ampicillin	1
pAGR309	Pcon-*cas9* + pBAD-λ Red genes + Ptet-gRNA-pBR322ori + p19-*infA*	oriR101	Ampicillin	This work; derived from pMP11
pAGR356	pgRNA-*lacZ*	pBR322	Chloramphenicol	This work; derived from pgRNA
pAGR357	pgRNA-*yraK/rsmI* intergenic region	pBR322	Chloramphenicol	This work; derived from pgRNA
pAGR358	pgRNA-*rhtBC* intergenic region	pBR322	Chloramphenicol	This work; derived from pgRNA

pAGR381	pgRNA-*exo*/*cea* intergenic region	pBR322	Chloramphenicol	This work; derived from pgRNA
pAGR300	pgRNA-*torST* intergenic region	pBR322	Chloramphenicol	This work; derived from pgRNA
pAGR278	pgRNA-*infA*	pBR322	Chloramphenicol	This work; derived from pgRNA
pAGR420	pgRNA-*recA*	pBR322	Chloramphenicol	This work; derived from pgRNA
pAGR421	pgRNA-*polB*	pBR322	Chloramphenicol	This work; derived from pgRNA
pAGR422	pgRNA-*dinB*	pBR322	Chloramphenicol	This work; derived from pgRNA
pAGR423	pgRNA-*umuDC*	pBR322	Chloramphenicol	This work; derived from pgRNA
pAGR295	p19-*gfpmut3* carrier for integration into *torST*	pSC101	Kanamycin	This work
pAGR353	Ptet-*cas9* carrier for integration into *lacZ*	p15A	Spectinomycin	This work
pAGR354	Ptet-*cas9* carrier for integration into *yraK*/*rsmI*	p15A	Spectinomycin	This work
pAGR372	Ptet-*cas9* carrier for integration into *rhtBC*	p15A	Spectinomycin	This work
pAGR382	Ptet-*cas9* carrier for integration into *exo*/*cea*	p15A	Spectinomycin	This work
**aTc-inducible kill switches**				
pAGR351	Ptet-*cas9*	pSC101	Kanamycin	This work
pAGR319 **groL-1**	Ptet-pgRNA-*groL*-1 + Pcon-*tetR*	p15A	Spectinomycin	This work
pAGR320 **groL-2**	Ptet-pgRNA-*groL*-2 + Pcon-*tetR*	p15A	Spectinomycin	This work
pAGR317 **ile-1**	Ptet-pgRNA-*ile*-1 + Pcon-*tetR*	p15A	Spectinomycin	This work
pAGR318 **ile-2**	Ptet-pgRNA-*ile*-2 + Pcon-*tetR*	p15A	Spectinomycin	This work
pAGR316 **rrs-1**	Ptet-pgRNA-*rrs*-1 + Pcon-*tetR*	p15A	Spectinomycin	This work
pAGR315 **rrs-2**	Ptet-pgRNA-*rrs*-2 + Pcon-*tetR*	p15A	Spectinomycin	This work
pAGR378 **Initial 2-gRNA**	Ptet-pgRNA-*ile*-2 + Pcon-*tetR* + Ptet-pgRNA-*groL*-2	p15A	Spectinomycin	This work
pAGR384 **Optimized 2-gRNA**	Ptet(opt)-pgRNA-*ile*-2 + Pcon-*tetR* + Ptet(opt)-pgRNA-*groL*-2	p15A	Spectinomycin	This work; derived from pAGR378
pAGR385 **ABX-independent initial 2-gRNA**	Ptet-pgRNA-*ile*-2 + Pcon-*tetR* + Ptet-pgRNA-*groL*-2 + p07-*infA*	p15A	Spectinomycin	This work; derived from pAGR378
pAGR387 **ABX-independent optimized 2-gRNA**	Ptet(opt)-pgRNA-*ile*-2 + Pcon-*tetR* + Ptet(opt)-pgRNA-*groL*-2 + p07-*infA*	p15A	Spectinomycin	This work; derived from pAGR384
pAGR398 **ABX-independent single gRNA optimized initial 2-gRNA**	Ptet-pgRNA-*ile*-2 + Pcon-*tetR* + Ptet-pgRNA-*groL*-2 + p07-*infA* + Ptet(opt)-pgRNA-*rrs*-2	p15A	Spectinomycin	This work; derived from pAGR385

pAGR390<br>**ABX-independent *infA* optimized initial 2-gRNA**	Ptet-pgRNA-*ile*-2 + Pcon-*tetR* + Ptet-pgRNA-*groL*-2 + Pcon(opt)-*infA*	p15A	Spectinomycin	This work; derived from pAGR385
pAGR399<br>**ABX-independent gRNA optimized, optimized 2-gRNA**	Ptet-pgRNA-*ile*-2 + Pcon-*tetR* + Ptet-pgRNA-*groL*-2 + p07-*infA* + Ptet(opt)-pgRNA-*rrs*-2	p15A	Spectinomycin	This work; derived from pAGR387
pAGR400<br>**ABX-independent *infA* optimized, optimized 2-gRNA**	Ptet(opt)-pgRNA-*ile*-2 + Pcon-*tetR* + Ptet(opt)-pgRNA-*groL*-2 + Pcon(opt)-*infA*	p15A	Spectinomycin	This work; derived from pAGR387
pAGR401<br>**CRISPRks [Mut14]**	Ptet(opt)-pgRNA-*ile*-2 + Pcon-*tetR* + Ptet(opt)-pgRNA-*groL*-2 + Pcon(opt)-*infA* + Ptet(opt14)-pgRNA-*rrs*-2	p15A	Spectinomycin	This work; derived from pAGR400
pAGR402<br>**CRISPRks Mut32**	Ptet(opt)-pgRNA-*ile*-2 + Pcon-*tetR* + Ptet(opt)-pgRNA-*groL*-2 + Pcon(opt)-*infA* + Ptet(opt32)-pgRNA-*rrs*-2	p15A	Spectinomycin	This work; derived from pAGR400
**Temperature sensors and 2-input kill switches**				
pAGR414	PtlpA-*tlpA-gfpmut3*	p15A	Spectinomycin	This work
pAGR415	PtlpA-*tlpA*-gfpmut3*	p15A	Spectinomycin	This work
pAGR417<br>**Initial 2-input CRISPRks**	Ptet(opt)-pgRNA-*ile*-2 + Ptet(opt20)-pgRNA-*groL*-2 + Pcon(opt)-*infA* + Ptet(opt14)-pgRNA-*rrs*-2 + PtlpA-*tlpA36-tetR*	p15A	Spectinomycin	This work; derived from pAGR401
pAGR510<br>**[Optimized] 2-input CRISPRks [2AA-81]**	Ptet(opt)-pgRNA-*ile*-2 + Ptet(opt20)-pgRNA-*groL*-2 + Pcon(opt)-*infA* + Ptet(opt14)-pgRNA-*rrs*-2 + PtlpA-*tlpA36*- RBS(opt_2AA-81)*_tetR_ssrA(opt_*2AA-81*)*	p15A	Spectinomycin	This work; derived from pAGR417
pAGR511<br>**Optimized 2-input CRISPRks 2AA-333**	Ptet(opt)-pgRNA-*ile*-2 + Ptet(opt20)-pgRNA-*groL*-2 + Pcon(opt)-*infA* + Ptet(opt14)-pgRNA-*rrs*-2 + PtlpA-*tlpA36*- RBS(opt_2AA-333)*_tetR_ssrA(opt_*2AA-333*)*	p15A	Spectinomycin	This work; derived from pAGR417
pAGR512<br>**Optimized 2-input CRISPRks 3AA-208**	Ptet(opt)-pgRNA-*ile*-2 + Ptet(opt20)-pgRNA-*groL*-2 + Pcon(opt)-*infA* + Ptet(opt14)-pgRNA-*rrs*-2 + PtlpA-*tlpA36*- RBS(opt_3AA-208)*_tetR_ssrA(opt_*3AA-208*)*	p15A	Spectinomycin	This work; derived from pAGR417
pAGR513<br>**Optimized 2-input CRISPRks 3AA-271**	Ptet(opt)-pgRNA-*ile*-2 + Ptet(opt20)-pgRNA-*groL*-2 + Pcon(opt)-*infA* + Ptet(opt14)-pgRNA-*rrs*-2 + PtlpA-*tlpA36*- RBS(opt_3AA-271)*_tetR_ssrA(opt_*3AA-271*)*	p15A	Spectinomycin	This work; derived from pAGR417
pAGR514<br>**Optimized 2-input CRISPRks 3AA-359**	Ptet(opt)-pgRNA-*ile*-2 + Ptet(opt20)-pgRNA-*groL*-2 + Pcon(opt)-*infA* + Ptet(opt14)-pgRNA-*rrs*-2 + PtlpA-*tlpA36*- RBS(opt_3AA-359)*_tetR_ssrA(opt_*3AA-359*)*	p15A	Spectinomycin	This work; derived from pAGR417
**No gRNA Controls**				
pAGR129	Empty vector	p15A	Spectinomycin	This work
pAGR397	Pcon-tetR + Pcon(opt)-*infA*	p15A	Spectinomycin	This work
pAGR427	Pcon-tetR + Pcon(opt)-*infA*	p15A	Chloramphenicol	This work
**Intermediate carrier plasmids for transforming gRNA plasmids**				
pAGR377	Pcon-*tetR*	oriR101	Kanamycin	This work
pAGR389	Pcon-*tetR* + p19-*infA*	oriR101	Kanamycin	This work; derived from pAGR377
**Miscellaneous**				
pDD81	p10-*gfpmut3*	NG2	Gentamycin	2

Supplementary Table 6 Genetic parts used in this work, related to Methods.

Part	Nucleotide Sequence
gRNA Cas9 handle and terminator	gttttagagctagaaatagcaagttaaaataaggctagtccgttatcaacttgaaaaagtggcaccgagtcggtgctttttttgaagcttgggcccgaacaa<br>aaactcatctcagaagaggatctgaatagcgccgtcgaccatcatcatcatcatcattgagtttaaacggtctccagcttggctgttttggcggatgagaga<br>agattttcagcctgatacagattaaatcagaacgcagaagcggtctgataaaacagaatttgcctggcggcagtagcgcggtggtcccacctgacccc<br>atgccgaactcagaagtgaaacgccgtagcgccgatggtagtgtggggtctccccatgcgagagtagggaactgccaggcatcaaataaaacgaaa<br>ggctcagtcgaaagactgggcctttcgttttatctgttgtttgtcggtgaac
cas9 with RBS	tcagcaggacgcactgaccaggaggtacaatcaATGgataagaaatactcaataggcttagatatcggcacaaatagcgtcggatgggcggtgatc<br>actgatgaatataaggttccgtctaaaaagttcaaggttctgggaaatacagaccgccacagtatcaaaaaaaatcttataggggctcttttatttgacagt<br>ggagagacagcggaagcgactcgtctcaaacggacagctcgtagaaggtatacacgtcggaagaatcgtatttgttatctacaggagatttttcaaatg<br>agatggcgaaagtagatgatagtttctttcatcgacttgaagagtcttttttggtggaagaagacaagaagcatgaacgtcatcctatttttggaaatatagta<br>gatgaagttgcttatcatgagaaatatccaactatctatcatctgcgaaaaaaattggtagattctactgataaagcggatttgcgcttaatctatttggcctta<br>gcgcatatgattaagtttcgtggtcatttttgattgagggagatttaaatcctgataatagtgatgtggacaaactatttatccagttggtacaaacctacaatc<br>aattatttgaagaaaaccctattaacgcaagtggagtagatgctaaagcgattctttctgcacgattgagtaaatcaagacgattagaaaatctcattgctc<br>agctccccggtgagaagaaaaatggcttatttgggaatctcattgctttgtcattgggtttgacccctaattttaaatcaaattttgatttggcagaagatgctaa<br>attacagctttcaaaagatacttacgatgatgatttagataatttattggcgcaaattggagatcaatatgctgatttgtttttggcagctaagaatttatcagatg<br>ctattttactttcagatatcctaagagtaaatactgaaataactaaggctcccctatcagcttcaatgattaaacgctacgatgaacatcatcaagacttgact<br>cttttaaaagctttagttcgacaacaacttccagaaaagtataaagaaatcttttttgatcaatcaaaaaacggatatgcaggttatattgatgggggagcta<br>gccaagaagaattttataaatttatcaaaccaattttagaaaaaatggatggtactgaggaattattggtgaaactaaatcgtgaagatttgctgcgcaagc<br>aacggacctttgacaacggctctattccccatcaaattcacttgggtgagctgcatgctattttgagaagacaagaagacttttatccatttttaaaagacaat<br>cgtgagaagattgaaaaaatcttgacttttcgaattccttattatgttggtccattggcgcgtggcaatagtcgttttgcatggatgactcggaagtctgaagaa<br>acaattaccccatggaattttgaagaagttgtcgataaaggtgcttcagctcaatcatttattgaacgcatgacaaactttgataaaaatcttccaaatgaaa<br>aagtactaccaaaacatagtttgctttatgagtattttacggtttataacgaattgacaaaggtcaaatatgttactgaaggaatgcgaaaaccagcatttctt<br>tcaggtgaacagaagaaagccattgttgatttactcttcaaaacaaatcgaaaagtaaccgttaagcaattaaaagaagattatttcaaaaaaatagaat<br>gttttgatagtgttgaaatttcaggagttgaagatagatttaatgcttcattaggtacctaccatgatttgctaaaaattattaaagataaagatttttggataatg<br>aagaaaatgaagatatcttagaggatattgttttaacattgaccttatttgaagatagggagatgattgaggaaagacttaaaacatatgctcacctctttgat<br>gataaggtgatgaaacagcttaaacgtcgccgttatactggttggggacgtttgtctcgaaaattgattaatggtattagggataagcaatctggcaaaac<br>aatattagatttttgaaatcagatggttttgccaatcgcaattttatgcagctgatccatgatgatagtttgacatttaaagaagacattcaaaaagcacaagt<br>gtctggacaaggcgatagtttacatgaacatattgcaaatttagctggtagccctgctattaaaaaaggtattttacagactgtaaaagttgttgatgaattgg<br>tcaaagtaatggggcggcataagccagaaaatatcgttattgaaatggcacgtgaaaatcagacaactcaaaagggccagaaaaattcgcgagagc<br>gtatgaaacgaatcgaagaaggtatcaaagaattaggaagtcagattcttaaagagcatcctgttgaaaatactcaattgcaaaatgaaaagctctatct<br>ctattatctccaaaatggaagagacatgtatgtggaccaagaattagatattaatcgtttaagtgattatgatgtcgatcacattgttccacaaagtttccttaa<br>agacgattcaatagacaataaggtcttaacgcgttctgataaaaatcgtggtaaatcggataacgttccaagtgaagaagtagtcaaaaagatgaaaa<br>actattggagacaacttctaaacgccaagttaatcactcaacgtaagtttgataatttaacgaaagctgaacgtggaggtttgagtgaacttgataaagctg<br>gttttatcaaacgccaattggttgaaactcgccaaatcactaagcatgtggcacaaattttggatagtcgcatgaatactaaatacgatgaaaatgataaa<br>cttattcgagaggttaaagtgattaccttaaaatctaaattagtttctgacttccgaaaagatttccaattctataaagtacgtgagattaacaattaccatcatg<br>cccatgatgcgtatctaaatgccgtcgttggaactgctttgattaagaaatatccaaaacttgaatcggagtttgtctatggtgattataaagtttatgatgttcgt<br>aaaatgattgctaagtctgagcaagaaataggcaaagcaaccgcaaaatatttcttttactctaatatcatgaacttcttcaaaacagaaattacacttgca<br>aatggagagattcgcaaacgccctctaatcgaaactaatggggaaactggagaaattgtctgggataaagggcgagattttgccacagtgcgcaaagt<br>attgtccatgccccaagtcaatattgtcaagaaaacagaagtacagacaggcggattctccaaggagtcaattttaccaaaaagaaattcggacaagct<br>tattgctcgtaaaaaagactgggatccaaaaaaatatggtggttttgatagtccaacggtagcttattcagtcctagtggttgctaaggtggaaaaaggga<br>aatcgaagaagttaaaatccgttaaagagttactagggatcacaattatggaaagaagttcctttgaaaaaaatccgattgactttttagaagctaaagga<br>tataaggaagttaaaaaagacttaatcattaaactacctaaatatagtcttttttgagttagaaaacggtcgtaaacggatgctggctagtgccggagaatta<br>caaaaaggaaatgagctggctctgccaagcaaatatgtgaatttttatatttagctagtcattatgaaaagttgaagggtagtccagaagataacgaaca<br>aaaacaattgtttgtggagcagcataagcattatttagatgagattattgagcaaatcagtgaattttctaagcgtgttattttagcagatgccaatttagataa<br>agttcttagtgcatataacaaacatagagacaaaccaatacgtgaacaagcagaaaatattattcatttatttacgttgacgaatcttggagctcccgctgc<br>ttttaaatattttgatacaacaattgatcgtaaacgatatacgtctacaaaagaagttttagatgccactcttatccatcaatccatcactggtctttatgaaaca<br>cgcattgatttgagtcagctaggaggtgacggcagcggtgattataaagatgatgatgataaatga
p07	tttacggctagctcagtcctaggtataatgctagc
p10	tttacggctagctcagtcctaggtacaatgctagc
p19	ttgacagctagctcagtcctaggtataatgctagc
Ptet	tccctatcagtgatagagattgacatccctatcagtgatagagatactgagcaca
Ptet libraries	tccctatcagtgatagagattgaNNtccctatcagtgatagagatactgagcaca
gfpmut3 with RBS	aagtcggtgacagataacaggagtaagtaATGagtaaaggagaagaacttttcactggagttgtcccaattcttgttgaattagatggtgatgttaatgg<br>gcacaaattttctgtcagtggagagggtgaaggtgatgcaacatacggaaaacttacccttaaatttatttgcactactggaaaactacctgttccatggcc<br>aacacttgtcactactttgacttatggtgttcaatgcttttcaagatacccagatcatatgaaacggcatgactttttcaagagtgccatgcccgaaggttatgt<br>acaggaaagaactatatttttcaaagatgacgggaactataagacacgtgctgaagtcaagtttgaaggtgatacacttgttaatagaatcgagttaaaa<br>ggtattgattttaaagaagatggaaacattcttggacacaagttggaatacaactataactcacacaatgtatacatcatggcagacaaacaaaagaat

	ggaatcaaagttaacttcaaaattagacacaacattgaagatggaagcgttcaactagcagaccattatcaacaaaatactccaattggcgatggccct<br>gtccttttaccagacaaccattacctgtccacacaatctgccctttcgaaagatcccaacgaaaagagagaccacatggtccttcttgagtttgtaacagct<br>gctgggattacacatggcatggatgaactatacaaaaggcctgcagcaaacgacgaaaactacgcttaagtagcttaa
infA with RBS	ggttcaaattacggtagtgataccccagaggattagATGgccaaagaagacaatattgaaatgcaaggtaccgttcttgaaacgttgcctaataccatg<br>ttccgcgtagagttagaaaacggtcacgtggttactgcacacatctccggtaaaatgcgcaaaaactacatccgcatcctgacgggcgacaaagtgact<br>gttgaactgaccccgtacgacctgagcaaaggccgcattgtcttccgtagtcgctga
PtlpA-*tlpA* with RBS	gctgtcctggtgccctttttcgctaccctgtcacgtctttaatttgtttgttagttagtttatttgttggtttgtttgtgtataatataagcataccccttta tgcggagactg<br>acagATGcgtccggcgacatacgaaccagaacagattattgaagcagggctggccctgcaggctgaaggacggaatatcaccgggttcgcactac<br>gtaaccaggtgggtggcggcaatccgacacgtctccgccagatatgggacgaataccaggcttcacagagcacggtcgtcactgaacccgttgccga<br>gctgccagtggaagtggctgaagaagtgaaggccgtctccgccgcgctgtccgaacgcatcacccagctggcgacagaactgaatgacaaggcggt<br>ccgggctgcagaacgccgggttgcggaagtcacgcgtgctgccggtgaacagaccgcacaggcagagcgggagctggccgacgccgcgcagac<br>agtcgacgacctggaagaaaaactggatgaactgcaggacagatatgacagtttgacgctggcgctggagtcagaacgttcactgcgtcagcagcat<br>gatgtggagatggcccagctgaaagagcgtcttgcggccgctgaagagaatacccgtcagcgagaggaacggtatcaggagcagaagacagtgct<br>gcaggatgcgcttaatgcggagcaggcacagcacaaaaacacgcgggaagacctgcagaaacgactggagcaaatttctgccgaagctaatgcg<br>cgtacagaagaactgaagtctgaacgcgataaagtcaatactctccttacccgccttgaatcgcaggaaaatgcgctggcctcagaacgtcagcagca<br>tctggccacccgcgaaacgctgcagcaacgcctcgagcaggccatcgctgacacgcaggcgcgcgccggtgagattgcacttgaacgtgacagagt<br>cagcagcctcaccgcaaggctggaatcgcaggaaaaggcctcctcggagcaactggtgcgtatgggcagtgaaatagccagtctgacagagcgttg<br>cacacagctggaaaaccagcgtgatgatgcccgtctggagacgatgggggagaaagaaacggtcgcggcactgcgtggtgaggctgaagccctga<br>agcgtcagaaccagtcactgatggcggcgctttcaggcaataaacagaccggtggccagaatgcgtga
*tlpA** [3]	ATGcgtccggcgacatacgaaccagaacagattattgaagcagggctggccctgcaggctgaaggacggaatatcaccgggttcgcactacgtaa<br>ccaggtgggtggcggcaatccgacacgtctccgccagatatgggacgaataccaggcttcacagagcacggtcgtcactgaactcgttgccgagctg<br>ccagtggaagtggctgaagaagtgaaggccgtctccgccgcgctgtccgaacgcatcacccagctggcgacagaactgaatgacaaggcggtccg<br>ggctgcagaacgccgggttgcggaagtcacgcgtgctgccggtgaacagaccgcacaggcagagcgggagctggccgacgccgcgcagacagt<br>cgacgacctggaagaaaaactggttgaactgcaggacagatatgacagtttgacgctggcgctggagtcagaacgttcactgcgtcagcagcatgatg<br>tggagatggcccagctgaaagagcgtcttgcggccgctgaagagaatacccgtcagcgagaggaacggtatcaggagcagaggacagtgctgcag<br>gatgcgcttaatgcggagcaggcacagcacataaacacgcgggaagaccagcagaaacgactggagcaaatttctgccgaagctaatgcgcgtac<br>agaagaactgaagtctgaacgcgataaagtcaatactctccttacccgccttgaatcgcaggaaaatgcgctggcctcagaacgtcagcagcatctgg<br>ccacccgcgaaacgctgcagcaacgcctcgagcaggccatcgctgacacgcaggcgcgcgccggtgagattgcacttgaacgtgacagagtcagc<br>agcctcaccgcaaggctggaatcgcaggaaaaggcctcctcggagcaactggtgcgtatgggcagtgaaatagccagtctgacagagcgttgcaca<br>cagctggaaaaccagcgtgatgatgcccgtctggagacgatgggggagaaagaaacggtcgcggcactgcgtggtgaggctgaagccctgaagcg<br>tcagaaccagtcactgatggcggcgctttcaggcaataaacagaccggtggccagaatgcgtga
Pcon-*tetR*	aattgacggctagctcagtcctaggtacagtgctagctcacacaggaaaggcctcgATGtccagattagataaaagtaaagtgattaacagcgcatta<br>gagctgcttaatgaggtcggaatcgaaggtttaacaacccgtaaactcgcccagaagctaggtgtagagcagcctacattgtattggcatgtaaaaaat<br>aagcgggctttgctcgacgccttagccattgagatgttagataggcaccatactcacttttgccctttagaaggggaaagctggcaagattttttacgtaata<br>acgctaaaagttttagatgtgctttactaagtcatcgcgatggagcaaaagtacatttaggtacacggcctacagaaaaacagtatgaaactctcgaaaa<br>tcaattagcctttttatgccaacaaggtttttcactagagaatgcattatatgcactcagcgctgtggggcattttactttaggttgcgtattggaagatcaagag<br>catcaagtcgctaaagaagaaagggaaacacctactactgatagtatgccgccattattacgacaagctatcgaattatttgatcaccaaggtgcagag<br>ccagccttcttattcggccttgaattgatcatatgcggattagaaaaacaacttaaatgtgaaagtgggtcctaa
tetR RBS library	YcacWatYttgWgRagKttRgg
tetR RBS (2AA-81)	ccactatcttgtggaggttggg
ssrA degradation tag library (2AA)	gcagcgaacgatgaaaactacNNNNNNgccgct
ssrA degradation tag library (3AA)	gcagcgaacgatgaaaactacNNNNNNNNNgct
Optimal ssrA degradation tag (2AA-81)	gcagcgaacgatgaaaactaccctgtggccgct

Supplementary Table 7 Key resources.

REAGENT or RESOURCE	SOURCE	IDENTIFIER
**Bacterial and virus strains**		
E. coli DH10B	Invitrogen	EC0113
E. coli Nissle 1917 (plasmid free)	DSMZ	DSM 16700
E. coli Nissle 1917 (plasmid free) Δ*lacZ*::Ptet-*cas9*	This paper	sAGR429
E. coli Nissle 1917 (plasmid free) Δ*lacZ*::Ptet-*cas9* *rhtBC*::Ptet-*cas9*	This paper	sAGR430
E. coli Nissle 1917 (plasmid free) Δ*lacZ*::Ptet-*cas9* *rhtBC*::Ptet-*cas9* *agaI*/*rsmI*::Ptet-*cas9*	This paper	sAGR431
E. coli Nissle 1917 (plasmid free) Δ*lacZ*::Ptet-*cas9* *rhtBC*::Ptet-*cas9* *agaI*/*rsmI*::Ptet-*cas9* *exo*/*cea*::Ptet-*cas9* *torST*::p19-*gfpmut3*	This paper	sAGR489
E. coli Nissle 1917 (plasmid free) Δ*infA* Δ*lacZ*::Ptet-*cas9* *rhtBC*::Ptet-*cas9* *agaI*/*rsmI*::Ptet-*cas9* *exo*/*cea*::Ptet-*cas9* *torST*::p19-*gfpmut3*	This paper	sAGR514
E. coli Nissle 1917 (plasmid free) Δ*infA* Δ*recA* Δ*polB* Δ*dinB* Δ*umuDC* Δ*lacZ*::Ptet-*cas9* *rhtBC*::Ptet-*cas9* *agaI*/*rsmI*::Ptet-*cas9* *exo*/*cea*::Ptet-*cas9* *torST*::p19-*gfpmut3*	This paper	sAGR596
**Chemicals, peptides, and recombinant proteins**		
Agar	Sigma	A1296
Ampicillin sodium salt	Gold Biotechnology	A-301
Anhydrotetracycline HCl (aTc)	Sigma	37919
L-(+)-arabinose	Sigma	A3256
Bacto casamino acids	ThermoFisher	223050
$CaCl_2$ $2H_2O$	Sigma	C5080
Carbenicillin disodium salt	Sigma	C1389
Chloramphenicol	Gold Biotechnology<br>Fisher Scientific	C-105<br>AC227920250
Deoxynucleotide Set (dNTPs)	G-Biosciences	786-460
1,4-Dithiothreitol (DTT)	Sigma	3483-12-3
DpnI	New England BioLabs	R0176
Gentamycin sulfate	Gold Biotechnology	G-400
D-(+)-Glucose	Sigma	G8270

Glycerol	Fisher Scientific	BP229-1
Kanamycin monosulfate	Gold Biotechnology	K-120
KCl	Sigma	P5405
KH_2PO_4	Sigma	P9791
LB broth, Miller	VWR	J106
M9 minimal salts, 5X	Sigma	M6030
$MgCl_2$	Sigma	7786-30-3
$MgSO_4$ $7H_2O$	Sigma	63138
Na_2HPO_4	Sigma	S5136
NaCl	Sigma	S7653
NAD^+	Sigma	53-84-9
PEG-8000	Sigma	1546605
Phusion high-fidelity DNA polymerase	New England BioLabs	M0530
SapI	New England BioLabs	R0569
Spectinomycin dihydrochloride pentahydrate	Gold Biotechnology<br>Sigma	S-140<br>S4014
Streptomycin sulfate	Sigma	S6501
Sucrose	Sigma	S0389
T4 DNA ligase	New England BioLabs	M0202
T5 exonuclease	New England BioLabs	M0663
Taq DNA Ligase	New England BioLabs	M0208
Tris base	Sigma	77-86-1
**Experimental models: Organisms/strains**		
C57BL/6J mice	The Jackson Laboratory	000664
**Oligonucleotides**		
See Supplementary Tables 2, 4		
**Recombinant DNA**		
See Supplementary Tables 3, 5, 6		
**Other**		
14 mL round bottom tubes	Fisher Scientific	14-959-11B

96-deep well plates	Fisher Scientific	E951032808
96-well black assay microplates	Fisher Scientific	07-000-088
96-well clear round bottom assay microplates	Corning	353910
PCR reaction strips	Fisher Scientific	T3202N
Plastic feeding tubes 18ga x 38mm	Instech	FTP-18-38
PureLink Quick Plasmid Miniprep Kit	Invitrogen	K210011
Purina Conventional Mouse Diet	Purina	5K67
Zymoclean Gel DNA Recovery Kit	ZYMO Research	D4008

Supplemental References

1. Mehrer, C.R., Incha, M.R., Politz, M.C. & Pfleger, B.F. Anaerobic production of medium-chain fatty alcohols via a beta-reduction pathway. *Metab Eng* **48**, 63-71 (2018).
2. DeLorenzo, D.M., Rottinghaus, A.G., Henson, W.R. & Moon, T.S. Molecular Toolkit for Gene Expression Control and Genome Modification in Rhodococcus opacus PD630. *ACS Synth Biol* **7**, 727-738 (2018).
3. Piraner, D.I., Abedi, M.H., Moser, B.A., Lee-Gosselin, A. & Shapiro, M.G. Tunable thermal bioswitches for in vivo control of microbial therapeutics. *Nat Chem Biol* **13**, 75-80 (2017).
