# Supplementary material for: Genetically stable CRISPR-based kill switches for engineered microbes
Source: Nat Commun. 2022 Feb 3;13:672. doi: 10.1038/s41467-022-28163-5 (PMC8813983; doi:10.1038/s41467-022-28163-5)
Supplement: Supplementary file 2 — Reporting Summary [file 41467_2022_28163_MOESM2_ESM.pdf]

nature portfolio

Corresponding author(s): Tae Seok Moon
Gautam Dantas

Last updated by author(s): Dec 29, 2021

Reporting Summary

Nature Portfolio wishes to improve the reproducibility of the work that we publish. This form provides structure for consistency and transparency in reporting. For further information on Nature Portfolio policies, see our Editorial Policies and the Editorial Policy Checklist.

Statistics

For all statistical analyses, confirm that the following items are present in the figure legend, table legend, main text, or Methods section.

n/a	Confirmed	
☐	☒	The exact sample size (*n*) for each experimental group/condition, given as a discrete number and unit of measurement
☐	☒	A statement on whether measurements were taken from distinct samples or whether the same sample was measured repeatedly
☐	☒	The statistical test(s) used AND whether they are one- or two-sided<br>*Only common tests should be described solely by name; describe more complex techniques in the Methods section.*
☐	☒	A description of all covariates tested
☐	☒	A description of any assumptions or corrections, such as tests of normality and adjustment for multiple comparisons
☐	☒	A full description of the statistical parameters including central tendency (e.g. means) or other basic estimates (e.g. regression coefficient) AND variation (e.g. standard deviation) or associated estimates of uncertainty (e.g. confidence intervals)
☐	☒	For null hypothesis testing, the test statistic (e.g. *F*, *t*, *r*) with confidence intervals, effect sizes, degrees of freedom and *P* value noted<br>*Give P values as exact values whenever suitable.*
☒	☐	For Bayesian analysis, information on the choice of priors and Markov chain Monte Carlo settings
☒	☐	For hierarchical and complex designs, identification of the appropriate level for tests and full reporting of outcomes
☒	☐	Estimates of effect sizes (e.g. Cohen's *d*, Pearson's *r*), indicating how they were calculated

Our web collection on statistics for biologists contains articles on many of the points above.

Software and code

Policy information about availability of computer code

Data collection	Data was collected using Tecan i-Control 3.3.10 and GuavaSoft 2.7.
Data analysis	Data analysis was performed using Excel 2016, FlowJo_Vl0, Prism 9.1, and SnapGene 2.3.2

For manuscripts utilizing custom algorithms or software that are central to the research but not yet described in published literature, software must be made available to editors and reviewers. We strongly encourage code deposition in a community repository (e.g. GitHub). See the Nature Portfolio guidelines for submitting code & software for further information.

Data

Policy information about availability of data

All manuscripts must include a data availability statement. This statement should provide the following information, where applicable:
- Accession codes, unique identifiers, or web links for publicly available datasets
- A description of any restrictions on data availability
- For clinical datasets or third party data, please ensure that the statement adheres to our policy

All source data and plasmid maps have been deposited to Mendeley Data (DOI: 10.17632/dwhfp2ycyw.1). Any additional information is available from the Lead Contact upon request.

Field-specific reporting

Please select the one below that is the best fit for your research. If you are not sure, read the appropriate sections before making your selection.

- [x] Life sciences
- [ ] Behavioural & social sciences
- [ ] Ecological, evolutionary & environmental sciences

For a reference copy of the document with all sections, see nature.com/documents/nr-reporting-summary-flat.pdf

Life sciences study design

All studies must disclose on these points even when the disclosure is negative.

Sample size	Sample sizes for animal experiments, reporter assays, and viability assays were chosen based on our previous work (PMID: 30926240, PMID: 34767760) and the literature, and represent sample sizes routinely used for these methods. No sample size calculations were performed during the design of experiments. In vitro experiments were performed in biological triplicate. Mouse experiments were performed with groups of 4 or 6 mice, with groups split across two cages.
Data exclusions	No data was excluded from analysis.
Replication	All in vitro experiments were performed in biological triplicate. Results were reproducible across different days. Mouse experiments were performed with two cages per experimental group to ensure reproducibility. Multiple Independent mouse experiments were not performed for the same experiment.
Randomization	Colonies were randomly selected from transformation plates for in vitro and mouse experiments. Instead of allocating colonies into different experimental groups, each colony was put through all relevant experimental conditions for a given experiment. Mice were randomly split into experimental groups.
Blinding	Blinding was not necessary or possible for these experiments. Blinding was not performed for in vitro assays or in animal experiments because knowledge of treatment groups was required in order to perform the assays and experiments. In addition, all measurements were quantitative/objective and not prone to subjective judgment by investigators.

Reporting for specific materials, systems and methods

We require information from authors about some types of materials, experimental systems and methods used in many studies. Here, indicate whether each material, system or method listed is relevant to your study. If you are not sure if a list item applies to your research, read the appropriate section before selecting a response.

Materials & experimental systems

n/a	Involved in the study
[x]	[ ] Antibodies
[x]	[ ] Eukaryotic cell lines
[x]	[ ] Palaeontology and archaeology
[ ]	[x] Animals and other organisms
[x]	[ ] Human research participants
[x]	[ ] Clinical data
[x]	[ ] Dual use research of concern

Methods

n/a	Involved in the study
[x]	[ ] ChIP-seq
[ ]	[x] Flow cytometry
[x]	[ ] MRI-based neuroimaging

Animals and other organisms

Policy information about studies involving animals; ARRIVE guidelines recommended for reporting animal research

Laboratory animals	All mouse experiments were performed in female 8-week old C57BL/6 mice (Jackson Labs C57BL/6J, RRID:IMSR_JAX:000664). Mice were housed in a specific pathogen free barrier facility maintained by WUSM DCM at 30-70% humidity and 68-79°F under a 12:12 hour light:dark cycle. Mice were provided feed (Purina Conventional Mouse Diet (JL Rat/Mouse 6F Auto) #5K67) and water ad libitum.
Wild animals	This study did not involve wild animals.
Field-collected samples	This study did not involve samples collected from the field.
Ethics oversight	All mouse experiments were approved by the Washington University in Saint Louis School of Medicine Institutional Animal Care and Use Committee (Protocol number: 21-0160), and performed in AAALAC-accredited facilities in accordance with the National Institutes of Health guide for the case and use of laboratory animals.

Note that full information on the approval of the study protocol must also be provided in the manuscript.

Flow Cytometry

Plots

Confirm that:

- [ ] The axis labels state the marker and fluorochrome used (e.g. CD4-FITC).
- [ ] The axis scales are clearly visible. Include numbers along axes only for bottom left plot of group (a 'group' is an analysis of identical markers).
- [ ] All plots are contour plots with outliers or pseudocolor plots.
- [x] A numerical value for number of cells or percentage (with statistics) is provided.

Methodology

Sample preparation	Cell culture samples were diluted to a final OD600 of 0.005-0.01 in 200 µL filtered phosphate-buffered saline supplemented with 2 mg/mL kanamycin in 96-well assay microplates for analysis.
Instrument	Guava easyCyte HT Serial Number 6735145210
Software	All data was analyzed using FlowJo_V10
Cell population abundance	Populations were only gated by forward and side scatter to capture E. coli cells. Generally, ~95% of the events were within this gate.
Gating strategy	Cells were gated using FSC and SSC. Generally, an oval gate with approximate minimum/maximum FSC values of 20/600 and SSC values of 30/2000 was used. However, values were slightly adjusted by experiment to account for changes in the population due to growth phase and medium. No figure demonstrating the gating strategy was provided since the gate is not used to separate populations, but rather to select the entire population of cells and only exclude debris. A detailed description of the gating strategy is provided here and in the Methods.

- [ ] Tick this box to confirm that a figure exemplifying the gating strategy is provided in the Supplementary Information.
